# A Qualitative Analysis of Dental Photography in Orthodontics: The Patient's Perspective

**DOI:** 10.1155/2018/5418592

**Published:** 2018-07-30

**Authors:** Muhsin Çifter

**Affiliations:** Department of Orthodontics, Faculty of Dentistry, Istanbul University, Turkey

## Abstract

**Purpose:**

Dental photography is an essential part of orthodontic treatment. It is used during all stages, and many components can affect the image quality. During the procedure, attendants and the patient must often work together to obtain high-quality images. These aspects likely influence the patient's experience, which is important in today's healthcare services. This study qualitatively investigated the effects of dental photography procedures on the patient experience.

**Methods:**

This research used a qualitative approach that included both observational and interview methods. Twenty patients (16-20 years old) underwent dental photography for the first time at the initial stage of orthodontic treatment.

**Results:**

The lack of detailed information regarding the procedure and the appearance of the intraoral mirrors and retractors were primary causes of patient stress prior to the procedure. During the procedure, the mirrors and retractors caused pain for most patients. The inefficient designs and lack of compatibility between the items used were the primary reasons for patient complaints.

**Conclusions:**

Patients must be informed in advance and in detail about the procedure and the equipment to be used. Improved designs for the camera flash system and the intraoral equipment are needed to maximize both patient satisfaction and image quality.

## 1. Introduction

The effects of advances in digital recording technologies are visible in different sectors. Dentistry is an important example of digital dental photography (DDP), which has become an essential part of orthodontic treatments [1]. DDP enables clinicians to record key stages of treatment. It also contributes to the orthodontic discipline in aspects including communication with patients, self-check of specialists, treatment planning, and provision of the treatment for clinical research, education, and marketing purposes to increase the patient's motivation and cooperation during the process [1–5]. Medicolegal concerns are another aspect, and DDP protects both patients' and dentists' rights in possibly difficult circumstances [6].

In the orthodontic discipline, at least four extraoral and five intraoral photographs are recommended. For proper treatment planning and documentation, the extraoral photographs should show the patient's correct appearance, especially the natural smile; the intraoral photographs should show the complete dentition and occlusion. During the DDP process, various errors may originate from the practitioner, the patient, and the poor design of the equipment used [7]. If the causes of the errors are defined, proper solutions can be implemented. Therefore, three aspects of the procedure should be investigated: the patient, the practitioner, and the equipment.

The relevant literature on DDP primarily focuses on the technological aspects of the procedure and their possible influences on picture quality [1, 3, 5, 8]. No prior research has been identified regarding the components and their effects on the patient's experience. However, patient perception of the procedure has not been addressed to date but has great importance in today's healthcare approach. In addition, new instrument designs should be patient-centred to increase comfort and efficiency. In this study, a qualitative approach was adopted that included both observational and interview methods to investigate the effects of DDP procedures on the patient's experience. Based on the results, a number of recommendations were made to increase the patient-centeredness of the conventional DDP procedure.

## 2. Materials and Methods

This research was approved by the ethics committee of the Faculty of Dentistry of Istanbul University in accordance with the Declaration of Helsinki and was supported by the Scientific Research Projects Unit of Mimar Sinan Fine Arts University. Informed and voluntary written consent were obtained from all the subjects.

This study used a qualitative approach to understand patients' actual experiences during a regular dental photography procedure. The methodology used in this research consisted of two commonly used qualitative research methods in the social sciences: observation and interview.

The observation method is primarily used for exploratory purposes and allows researchers to directly obtain data about actions, behaviours, and real-life experiences [9, 10]. This method is also useful for the detection of unusual aspects [10], because in certain cases there could be a difference between what people say and what they do or experience [9]. However, the observation method is recommended as a supportive or supplementary method due to its disadvantages, including the observers' influence in the field and the considerable time necessary for the procedure and analysis [9, 10].

In this study, the observation method was used in conjunction with face-to-face, semistructured interviews [9] to collect in-depth insights from the study participants. The interview is a particularly useful method for the collection of information about people's experiences, attitudes, feelings, or reasons for discontent [11]. Therefore, semistructured interviews were used as the primary data collection method in this study and were supported by observation of the patients during their dental photography procedures.

### 2.1. Selection of the Participants

This study included 20 participants (10 males and 10 females). Patients were excluded if they were older or younger than 16-20 years of age, had disabilities, had temporomandibular joint problems, had cleft lip and/or palate anomalies, or had undergone a previous dental photography procedure. Due to these criteria, purposive sampling was considered the appropriate sampling method for this research [9].

To ensure anonymity, all participants were given a unique code (beginning with “A” for male and “B” for female participants) during analysis. Their interview responses are presented using these codes.

### 2.2. Study Setting and Procedures

**Figure 1:**
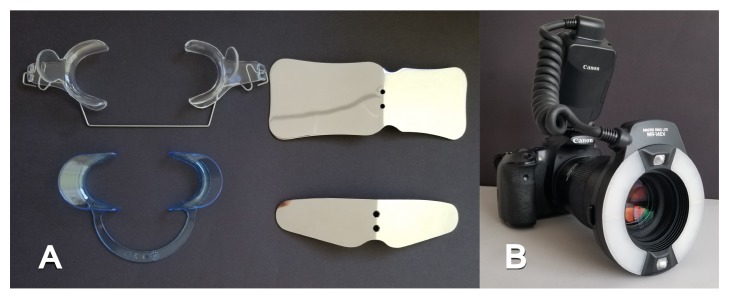
Primary equipment used during the procedure. (A) Spandex and wire type cheek and lip retractors and occlusal-buccal mirrors; (B) DSLR camera with Ring-Lite.

All observational and interview studies were performed in the Orthodontics Department of Istanbul University; the overall research methodology could be considered a contextual inquiry [12]. To ensure reliable results from all participants, the photography procedure was standardised as previously described [13, 14]. All photographs were taken by the same professional technician who was in charge of this procedure for the department. A Canon EOS 60D digital single lens reflex (DSLR) camera with a 100-mm Macro Lens and a Canon MR-14EX Macro Ring-Lite (Canon, Tokyo, JP) was used for photography (Figure 1).

**Figure 2:**
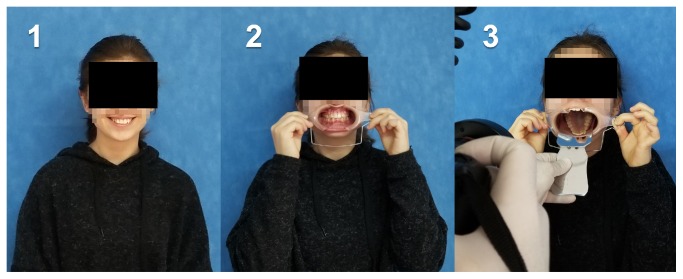
Stages of digital dental photography. Each image includes the respective stage number.

The process of dental photography was investigated in three stages: portrait and profile photographs; intraoral frontal and profile photographs; and intraoral buccal and occlusal photographs. A plain, coloured background was used in stage 1; spandex (Hager & Werken, Duisburg, DE) and wire type cheek retractors (Masel, Bristol, PA) were used in stage 2; and both retractors and dental mirrors (Ortho Technology, Florida, USA) were used in stage 3 (Figure 2). A bowl of hot water was used to warm the mirrors to avoid fogging, which is a commonly used method among clinicians. All of these items are likely to influence the patient experience at certain points during the procedure.

### 2.3. Data Analysis

Data analysis included the analysis of video recordings gathered from observations and data collected from semistructured interviews. Seventeen of 20 participants permitted video recording of their dental photography sessions. All 20 participants agreed to voice recording during the interviews. All data derived from the interviews were transcribed verbatim, and thematic analysis was performed using QSR NVivo 11 Qualitative Data Analysis Software (ORS International, Victoria, AU). Thematic analysis included the coding of raw data and categorization under potential themes to construct thematic networks to interpret new meanings [9, 11]. This method enabled researchers to acquire an in-depth understanding of patients' feelings about the procedure and the products used. In vivo coding, which refers to the use of words and short phases directly from participants' own expressions, was applied during analysis [15].

## 3. Results

### 3.1. Observations

In all, 10 male and 10 female participants were observed. However, 3 participants did not agree to a video recording of their sessions. During the analysis, commonly observed procedural problems that were likely to affect patient experience were recorded regardless of their frequency and were categorized; they are summarized in Table 1.

Many procedural difficulties had a direct effect on patient satisfaction. Patient stress, difficulties in equipment positioning and control, nonadjustable standard equipment sizes, lack of communication, and saliva accumulation were frequently observed problems.

### 3.2. Interviews

The interviews consisted of questions in two primary parts: the patients' thoughts regarding (1) their overall dental photography experience and (2) their reflections on each stage of the procedure.

#### 3.2.1. Overall Experience

Two important findings of the patients' overall experience were derived during the interviews. First, 40% (8 of 20) of patients were pleased that their orthodontic treatment had begun and were positively motivated. They stated that the procedure was for their well-being and aesthetic appearance; therefore, any difficulty was worthwhile.

- My feelings are positive because the procedure was for my well-being. My clinician wanted my teeth to be photographed, so I had to have it done (*A*8).

Second, 75% (15 of 20) of patients expressed that they received very little or no information regarding the procedure prior to the procedure. Some patients also indicated that they were misinformed by other people who had undergone a similar procedure, and therefore they felt unnecessarily stressed. Three participants also mentioned that the actual procedure was easier than they expected.

- I spoke with several people who had undergone this treatment. They told me that the procedure would make me sick, so I was initially apprehensive. But it was not as bad as the stories (*B*5).

- I did not receive any information on what the procedure would be like. I was expecting something similar to an X-ray scan (*B*4).

A number of patients also reported that they experienced intrinsic problems during the procedure due to feelings of nervousness or discomfort caused by the intraoral equipment. Physical pain was also reported to be an important influence on their overall experience. In addition, three patients reported that the technician's behaviour affected their experience.

- I didn't like that someone directs you to ‘turn left' and ‘turn right', and then puts weird things in your mouth. I didn't like it (*B*3).

#### 3.2.2. Experience during Different Stages of the Procedure

The results suggest that, during each stage of the procedure, participants experienced diverse problems that also influenced their overall experience. With the exception of the first stage, it was found that the equipment used had the largest impact.

While taking portrait and profile photographs, the patients reported that privacy was their primary concern. For example, two of the patients were asked to remove their head scarves during the procedure to obtain a complete facial view; both patients expressed their discomfort regarding this requirement. Three participants said that smiling on command was challenging. This finding was also evident during the observational study, and the patients indicated that nervousness hindered their ability to perform this simple emotional expression.

- When he (the technician) took my profile photograph, I felt like a criminal (*B*1).

-I don't like people taking my photograph, and I have avoided this for some time. For this reason, I thought it was a bit weird (*A*7).

During the extraoral close-up photography stage, the retractors were reported to be the primary source of discomfort. In all, 70% (14 of 20) of patients reported different problems (39 coding references), which could be divided into the following three categories.

*Physical Discomfort due to Use of the Retractors on Themselves*. Nine patients reported physical discomfort due to the retractors, including pain, irritation, and strain on the mouth tissue. This was the most frequently expressed problem category during intraoral frontal close-up photography.

-I think the retractor could be smaller. It is very big and strains your mouth. Maybe a smaller retractor would work better and would be more comfortable; with this one, you need to open and stretch your mouth too much, and it forces your jaw (*A*3).

- The retractors hurt my mouth a bit, but everything was alright (*B*8).

*Ideational Discomfort about Using the Retractors on Themselves*. Six patients reported discomfort associated with the negative feeling they had while using the retractor. These feelings were expressed as nervousness, weirdness, and anxiety.

- I felt embarrassed while the retractor was in my mouth because I think my teeth look bad. I don't know, maybe it just looks weird, but it was not a good feeling (*B*2).

*Prejudice about the Retractors' Appearance*. Four patients reported that they felt stressed when they had first seen the retractor, as they could not understand its function or thought it might hurt their mouth. One patient argued that the appearance of retractors could scare children and suggested designing them in a colourful way.

-If they use the same thing on children, it may scare them. Something colourful may be better (*A*5).

- I thought the mirror would cut my cheeks, but it was not that disturbing (*B*1).

Both retractors and dental mirrors were used during the stage of intraoral close-up buccal and occlusal photography. The results of the interviews suggested that dental mirrors were the primary source of discomfort during this stage. In total, 17 of 20 patients reported problems (30 coding references), which were categorized under the same themes as the retractors. However, their weights were different.

*Prejudice about the Mirrors' Appearance*. This was the most frequently mentioned category during intraoral photography; 7 patients reported 15 coding references. The primary feelings reported were anxiety, surprise, and fear when they first saw the dental mirrors. The patients reported that they could not understand the purpose of the equipment and believed they might hurt themselves during the procedure.

-I did not have concerns at the beginning. I was thinking it would be simple: I smile and the technician takes my photograph. Then, when I saw the metal things (mirrors), I felt a bit stressed because I wondered if they would hurt. But the experience was not that bad (*A*9).

*Physical Discomfort regarding the Use of the Mirrors on Themselves*. Six patients reported physical discomfort regarding the mirrors, such as pain and irritation of the intraoral soft tissues. However, more distinctly than the retractors, patients also expressed that they felt nausea due to the mirrors inside their mouths, complained about the temperature of the mirrors, and had difficulty breathing through the nose while the mirrors were inside their mouths.

-I did not have any problem with the big mirror [occlusal mirror], but the one used on the sides put pressure on my gums (*B*10).

- One of the mirrors was very hot. I would have appreciated if it had been cooler before putting it in my mouth (*A*9).

- When there is something in my mouth, I feel sick because I cannot swallow, and I had that feeling (*A*10).

*Ideational Discomfort about Using the Mirrors on Themselves*. Two patients reported discomfort associated with negative feelings while using the mirrors on themselves. These feelings were expressed as weirdness or embarrassment due to their appearance while the mirrors were used inside their mouths.

- I felt weird about the mirrors. They did not hurt, but this was the first time I had experienced something like this (*B*4).

In addition, seven patients complained about the pain caused by the buccal mirrors during the procedure. Pressure, pain, and strain on the gingival tissues and lips were reported by patients during the interviews. Three patients also reported that the occlusal mirror did not fit comfortably inside their mouth due to its size.

Finally, 45% (9 of 20) of the participants complained about the ring flash burst in their eyes. During the intraoral photography stage, ring flashes attached to the camera are widely used to capture clear macro images without shades. However, the patients expressed their feelings about flash burst with expressions such as direct flash burst in the eyes, very close distance for a flash burst, lacrimation due to the light, tiredness of eyes due to the light, discomfort due to too much light, and reflexive blink due to the light.

## 4. Discussion

Proper documentation is essential for orthodontic treatments, and DDP is a fundamental and widely preferred component of clinical documentation [1–6]. Until the intervention of digital photography, the images taken by conventional cameras could only be visible after photographic processing. Digital photography enables displaying the captured images instantly which gives the clinician the opportunity to capture the ideal images without patient call-backs. The other essential advantages of digital photography are the ease of transmitting the images, opportunity of detailed editing, and ease of archiving. In orthodontic and dentofacial treatments the patient's intraoral and extraoral appearance may be dramatically changed so high-quality photographs are mandatory for documenting these chances. Also for treatment planning and medico-legal necessity pretreatment malocclusion and soft and hard tissue health conditions should also be visually documented in detail. Basically clinical photography is composed of three main aspects: the photographer, the patient, and the equipment. Existing researches on this field are mostly focused on used methods, getting high-quality images, and encountered technical difficulties. However patient's perspective is a key aspect to improve the clinical photography outcomes and for a patient-centred medical approach. In the present study, it was determined that the mirrors used for occlusal and buccal intraoral photography and the retractors were the most complained-about items used in the procedure and have an overall impact on the patient's experience.

In this study, 40% of the patients were motivated before the procedure, as this was the first step of their treatment and they were willing to start. However, 75% of the patients reported that they were misinformed about the procedure which, according to the interviews, caused stress. In addition, signs of stress were observed before the procedure due to the appearance of the retractors and the mirrors. Another issue related to the stress and anxiety of the patients was observed during the smiling photographs. Full-face, naturally smiling photographs are essential for treatment planning, especially for patients who require improvement in gingival and incisor appearance for an aesthetic smile [8, 16]. Many patients failed to achieve a simple natural smile due to the stress induced by the procedure, privacy issues, and malocclusion-related embarrassment because of their appearance. Based on these data, it can be inferred that patient stress can be considerably reduced if adequate information regarding the procedure and the instruments used is provided before the procedure. In addition, visually appealing designs for the retractors and mirrors may help reduce patient anxiety.

Observations have shown that the intraoral photography stage was the most challenging for both the technician and the patients. Most patient complaints were related to the retractors and the intraoral mirrors. Large and durable retractors are needed to take a photograph of the desired intraoral field of view. However, the patients reported that the size and structure of the retractors primarily caused pain and a feeling of excessive strain on the soft tissues. In addition, the size and appearance of the equipment gave the patients the impression that the equipment would not fit in their mouth easily, which was also a cause of anxiety. These problems were more prominent for the mirrors, which were the most complained-about items in the procedure. Acquisition of the buccal and occlusal image of the distal aspect of the second molars was a challenge for both the technician and the patients. Due to the inadequate design of the buccal mirrors, soft tissue irritation and pain were the primary complaints. Feelings of nausea and difficulty breathing through the nose to minimise fog accumulation were other concerns raised by the patients regarding the occlusal mirror. The heat of the mirrors, intended to prevent fog accumulation, was another complaint of some patients.

During the intraoral buccal and occlusal photography stage, three different items are used simultaneously (camera, retractors, and mirrors). Several problems were encountered due to the incompatibility of the items; these issues affected the success of the procedure and the comfort of the patients. However, in this study, the technician was unassisted during the procedure. The technician needed to manage the orientation of the patients' head, the mirrors, and the retractors with one hand, while trying to capture the appropriate image using a heavy camera with the other. Patient assistance was inevitable because the occlusal mirror became stuck between the retractors, and the orientation of the patient's head changed as a result. In addition, the technician was required to adopt challenging postures, as he was attempting to acquire the best image while also trying to hold the mirrors (buccal and occlusal) in position without disturbing the patient. These problems occurred because the items in use were not designed to be used together; they were not adjustable and so were incompatible with each other. Problems of incompatibility and the need for assistance from patients can be eliminated by better design solutions. The items being used should be compatible with each other and should reduce the need for patient assistance without increasing the technician's workload.

Saliva accumulation was another important concern of intraoral photography. Lack of aspiration of saliva without interrupting the field of view was a challenge for the technician and a disturbance for the patients. From these results, it can be clearly said that practice and patient-friendly designs are necessary to improve both the technical procedure and patient comfort.

An issue that has not been previously mentioned in this field is the influence of the flash burst on the patient. In this study, 45% of the patients complained about the ring flash burst in their eyes from a close distance. The primary concerns were lacrimation, eye tiredness, and reflexive blink due to the severe light. As the working distance (distance between the subject and the camera) was as low as 20-30 cm for intraoral images, the flash burst inevitably affected patients' eyes unfavourably. These complaints should be addressed by new designs, or special eyeglasses may be worn to prevent the undesirable effects of the bright light.

**Figure 3:**
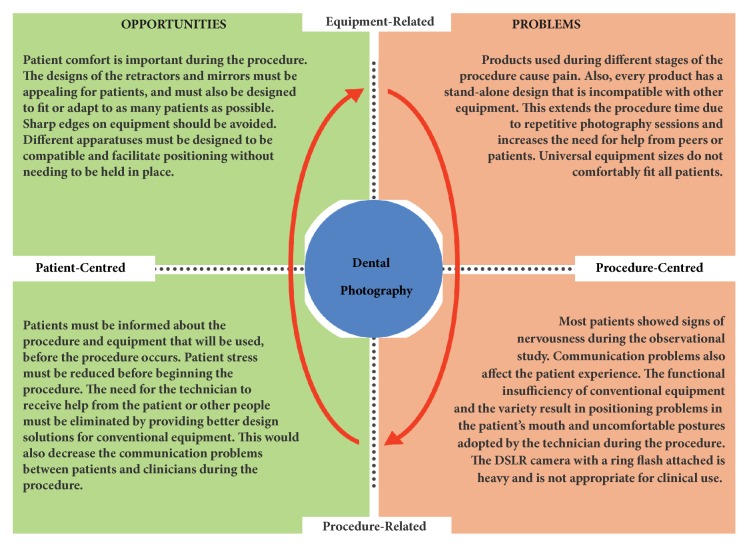
Primary problems and opportunities related to digital dental photography.

Figure 3 summarizes the results of this research in two dimensions as the equipment/procedure-related and patient/procedure-centred aspects of DDP. It also highlights the problems observed and suggests opportunities to provide better patient experiences.

The small sample size was a limitation of this research. However, this was a qualitative study and substantial time was required to collect and analyse the observational and interview data. In addition, this study enabled the collection of important insights of patients regarding the actual procedure and the identification of key factors that are likely to affect the patient's experience and overall motivation for treatment. Future research should include the development of a specific questionnaire to assess the impact of these factors on the patient's experience, which will enable data collection from a larger sample group and acquisition of generalizable results.

## 5. Conclusions

Patient discomfort and the technician's need for additional assistance for this simple procedure are due to design- and usability-related shortcomings of the equipment used during the procedure. These items can also cause pain and anxiety in patients due to inadequate design.

1. Patient stress prior to DDP can be reduced by providing detailed information about the procedure and the items to be used.
2. The retractors and the mirrors were the most painful and complained-about items. New designs are needed to improve patient comfort.
3. The items used during the procedure should be more compatible with each other to reduce technician effort and maximize patient comfort.
4. To protect the patient's eyes from the flash burst, special glasses can be used. New flash systems designed specifically for dental photography are needed to reduce patient complaints and increase patient comfort.

## Figures and Tables

**Table 1:** Common problems that were likely to affect the patient's experience and the procedural efficiency.

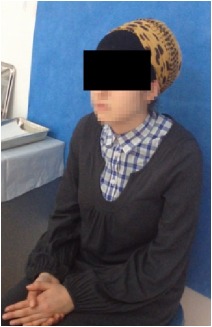	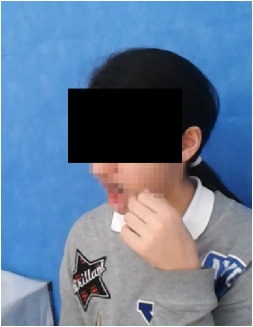	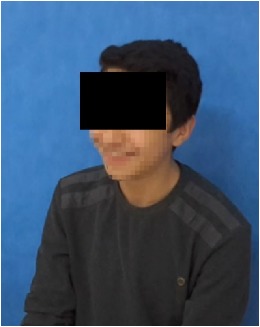	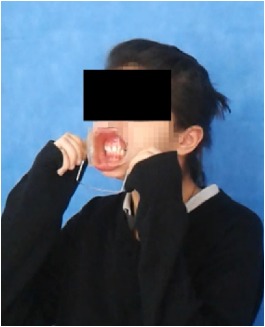

Several patients showed signs of stress while waiting for the photography procedure to start.	Several patients seemed uneasy when they observed the equipment to be used, particularly the dental mirrors. Some patients verbally expressed their impressions accordingly.	The patients were asked to smile in order to capture photographs of their natural, full-face, smiling expressions. However, many patients failed to provide this simple facial expression due to stress, which resulted in additional shootings and extended the procedure.	The size of the standard equipment did not fit some patients comfortably.

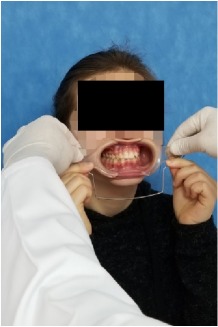	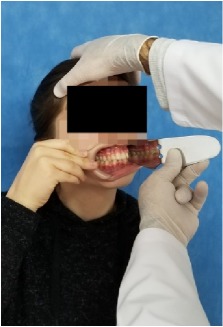	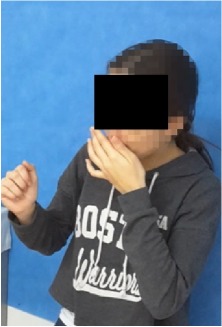	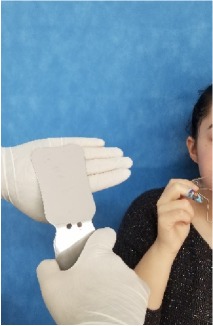

Due to the amount of equipment that required simultaneous use, patients were asked to help with positioning. In certain cases, patients could not understand what they were asked to do, which extended the procedure.	Due to equipment positioning and communication problems, in some cases the technician was required to physically touch and manipulate the patient's head.	Saliva accumulation was a frequent problem, and was observed in most patients.	During intraoral photography, the clinician used hot water to warm the metal mirrors to prevent fogging due to patient breathing. He determined the safety of the mirror's temperature using his gloved hand.

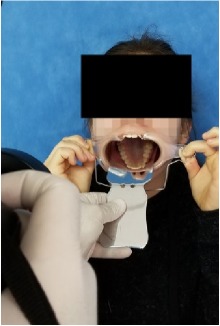	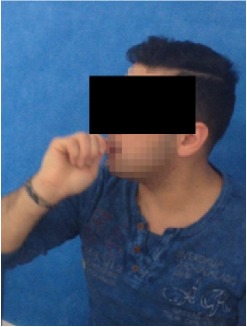	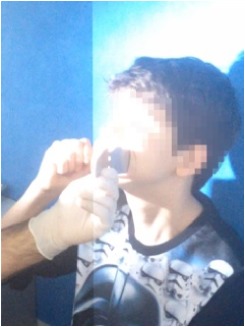	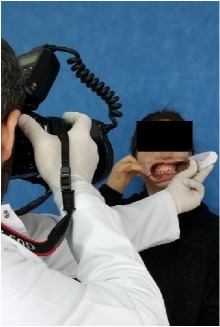

The occlusal mirror lodged between the retractors during intraoral occlusal photography of some patients. If that occurred, the patient's head position changed as the technician withdrew the mirror.	It was observed that patients' intraoral soft tissues hurt during certain stages of the procedure; pain was identified by the patient's facial expression. One patient also verbally expressed this problem during his procedure.	During the intraoral and extraoral photograph stages, it was observed that the flash bursts from a very close distance hurt some patients' eyes.	The clinician required patients to adopt uncomfortable postures due to the range of equipment he was required to concurrently position and control. He was also required to hold the heavy camera with a ring flash attached with only one hand.

## Data Availability

All the data regarding the results of this research are generated during the study.
